# Thickness distribution and site-specific variability of thermoformed multilayer clear aligners: an *in vitro* study

**DOI:** 10.3389/fdmed.2026.1863922

**Published:** 2026-06-01

**Authors:** Thi Bich Van Tran, Ngoc Anh Thu Pham

**Affiliations:** Faculty of Dentistry, University of Medicine and Pharmacy at Ho Chi Minh City, Ho Chi Minh City, Vietnam

**Keywords:** aligner thickness variation, clear aligner, in-house aligner production, thermoformed aligners, thickness distribution analysis

## Abstract

**Introduction:**

Thermoforming is a critical step in the fabrication of clear aligners and is associated with non-uniform deformation of thermoplastic materials, resulting in non-uniform thickness distribution. Because aligner thickness directly influences stiffness and force delivery, such variability may affect the accuracy and predictability of orthodontic treatment.

**Aim:**

This study aimed to evaluate the thickness distribution of thermoformed clear aligners across different tooth regions and anatomical sites, with a focus on identifying patterns of non-uniform material thinning.

**Methods:**

Ten thermoformed aligners fabricated from 760 µm multilayer thermoplastic sheets (Zendura FLX) were analyzed *in vitro*. Thickness measurements were obtained using a calibrated digital caliper at standardized anatomical reference points across anterior, canine, premolar, and molar regions. Generalized estimating equations (GEE) with robust standard errors were used to account for repeated measurements within the same aligner, and pairwise comparisons were adjusted using the Bonferroni correction.

**Results:**

Significant differences in thickness were observed among tooth regions (*p* < 0.001), with the lowest values in anterior teeth (404.0 ± 5.9 µm) and the highest in molars (481.6 ± 6.0 µm). Thickness reduction ranged from approximately 36% to 47% relative to the original material. Site-specific analysis demonstrated that the facial regions generally exhibited lower thickness values than corresponding palatal regions, indicating non-uniform thermoforming-induced deformation patterns. These findings demonstrated that anatomical location was a major determinant of thickness variability.

**Conclusion:**

Thermoformed clear aligners exhibit pronounced and systematic non-uniform thickness distribution strongly influenced by tooth morphology and local geometry characteristics. This non-uniform thickness distribution may contribute to variations in force delivery and should be considered in aligner design and clinical application.

## Introduction

Clear aligner therapy (CAT) has become a widely adopted modality in contemporary orthodontics due to its advantages in aesthetics, patient comfort, and removability compared with conventional fixed appliances (1, 2). Despite these benefits, clinical outcomes remain variable, with reported discrepancies between predicted tooth movement from digital treatment planning and actual clinical results. In particular, previous studies demonstrated that forces and moments delivered by aligners *in vivo* often differ from those estimated in virtual simulations, highlighting persistent limitations in biomechanical predictability (3–5).

A key factor contributing to this variability is the thickness distribution of the aligner. During thermoforming, a heated thermoplastic sheet is stretched over a dental model and undergoes non-uniform deformation as it adapts to complex dental geometry. This process leads to localized thinning, which may reduce material stiffness, alter force magnitude, and compromise durability (6, 7). Given that aligner stiffness is highly sensitive to thickness, even minor variations may result in disproportionate changes in force delivery, thereby affecting treatment efficiency and predictability. Previous investigations have primarily focused on single-layer thermoplastic materials, particularly polyethylene terephthalate glycol (PET-G), consistently demonstrating significant thickness reduction after thermoforming, especially in high-curvature regions such as cervical margins and incisal edges (6, 7). However, these studies often assessed global thickness changes or limited anatomical areas, and detailed characterization across different tooth types and surface locations remains limited. More recently, multilayer or hybrid materials have been introduced to improve mechanical performance. However, the thermoforming behavior of such materials is more complex, and current evidence regarding their thickness distribution remains insufficient. In addition, thickness variation is not uniform but depends on tooth morphology and anatomical location, potentially leading to uneven force systems across the dental arch. Despite its clinical relevance, a comprehensive understanding of thickness distribution across both tooth regions and anatomical sites remains lacking.

Therefore, the present *in vitro* study aimed to evaluate the thickness distribution of thermoformed clear aligners, with a focus on tooth-specific and site-specific variability.

## Methods

**Figure 1:**
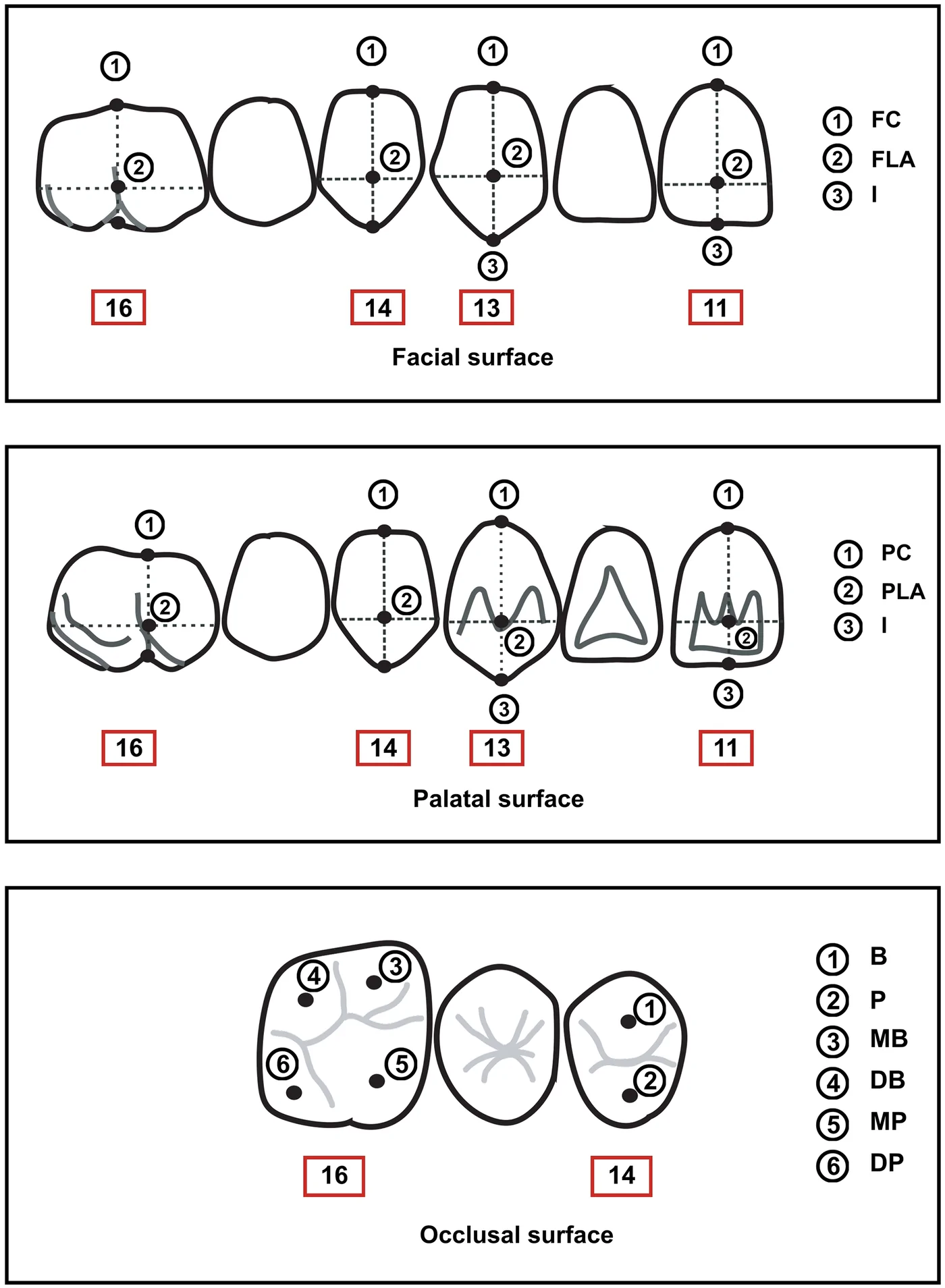
Illustration of anatomical reference points used for aligner thickness measurements.

This *in vitro* study evaluated the thickness distribution of thermoformed clear aligners fabricated from Zendura FLX thermoplastic sheets with an initial thickness of 760 µm. A total of ten aligners were produced under standardized thermoforming conditions using a pressure-forming unit (Drufomat Scan, Dreve, Germany) (Figure 1). All specimens were trimmed at the gingival margin by a single trained operator to ensure procedural consistency.

Thickness measurements were obtained using a calibrated digital caliper (Panme Syntek, China) with a resolution of 0.001 mm. The caliper was equipped with pointed measuring tips. To ensure reproducibility and anatomical consistency, measurements were performed at predefined reference points across representative maxillary teeth, including the central incisor (tooth 11), canine (tooth 13), first premolar (tooth 14), and first molar (tooth 16). These reference points were defined based on anatomical landmarks and standardized axes to allow consistent positioning of the measurement probe.

**Figure 2:**
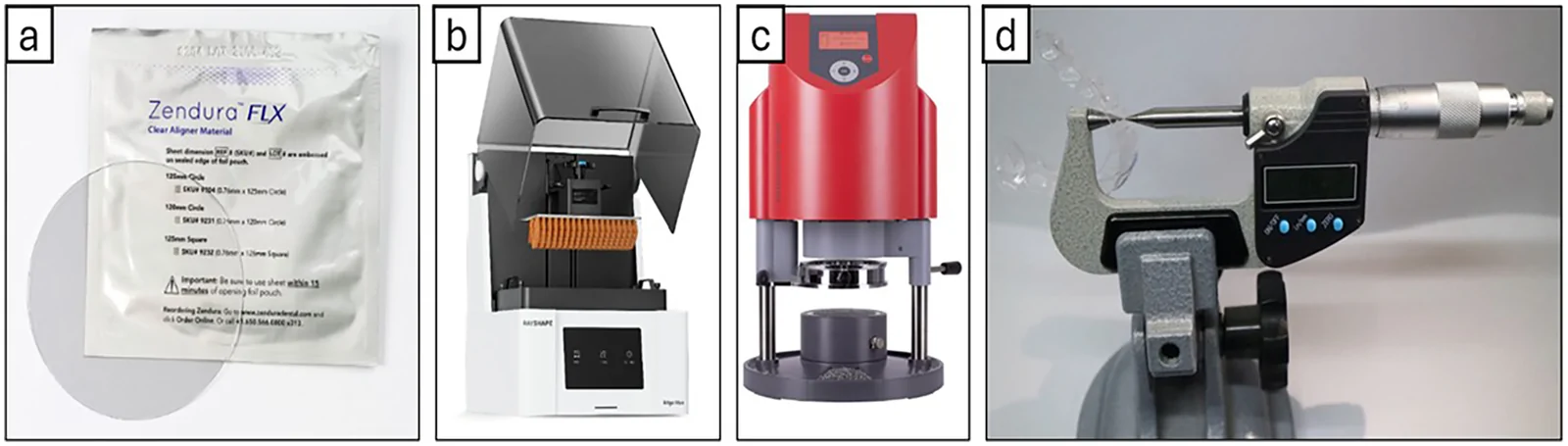
Materials and equipment used for aligner fabrication and thickness measurement: **(a)** thermoplastic sheets (zendura FLX); **(b)** 3D printer (rayshape edge E2); **(c)** thermoforming machine (drufomat scan); **(d)** digital caliper (panme syntek).

The anatomical reference points used for thickness measurements are summarized in Table 1 and illustrated in the Figure 2. Briefly, measurements in anterior and canine teeth were obtained at five locations, including the incisal edge, facial long-axis, facial cervical, palatal long-axis, and palatal cervical points. In posterior teeth, additional measurements were recorded at cusp tips to account for occlusal morphology. For premolars, buccal and palatal cusp tips were included, whereas for molars, measurements were taken at mesiobuccal, distobuccal, mesiopalatal, and distopalatal cusp tips. These cusp-based measurements were further aggregated to represent the occlusal surface.

**Table 1:** Anatomical reference points used for aligner thickness measurements.

Reference point	Definition	Anatomical region	Assessed teeth
I	Most coronal central point of the incisal or cusp-tip region	Incisal	11, 13
FLA	Long-axis point of the facial surface	Facial	11, 13, 14, 16
FC	Most cervical point of the facial surface	Facial-cervical	11, 13, 14, 16
PLA	Long-axis point of the palatal surface	Palatal	11, 13, 14, 16
PC	Most cervical point of the palatal surface	Palatal-cervical	11, 13, 14, 16
B	Buccal cusp tip	Cusp tip (OS)	14
P	Palatal cusp tip	Cusp tip (OS)	14
MB	Mesiobuccal cusp tip	Cusp tip (OS)	16
DB	Distobuccal cusp tip	Cusp tip (OS)	16
MP	Mesiopalatal cusp tip	Cusp tip (OS)	16
DP	Distopalatal cusp tip	Cusp tip (OS)	16

For premolars, occlusal surface (OS) values were derived from pooled buccal (B) and palatal (P) cusp-tip measurements. For molars, OS values were derived from pooled mesiobuccal (MB), distobuccal (DB), mesiopalatal (MP), and distopalatal (DP) cusp-tip measurements.

Each measurement was performed twice with the caliper probe oriented perpendicular to the aligner surface. When discrepancies between repeated measurements exceeded 20 µm, a third measurement was obtained, and the median value was recorded. All measurements were conducted by a single operator, and intra-operator reliability was assessed through repeated measurements on a subset of samples. The operator underwent calibration training under the supervision of an experienced metrology specialist. Intra-operator reliability and agreement with the reference examiner were evaluated by repeated measurements of all 10 aligners (24 sites per aligner) after a 2-week interval. Reliability was assessed using intraclass correlation coefficients (ICC), which demonstrated excellent reliability (ICC = 0.91). To minimize operator fatigue, only one aligner was measured per day.

Statistical analysis was performed using Generalized Estimating Equations (GEE) with robust standard errors to account for correlated repeated measurements obtained from the same aligner. The GEE model was specified with a Gaussian distribution, identity link function, and exchangeable correlation structure. Overall differences among tooth regions and anatomical sites were evaluated using Wald chi-square tests. Pairwise comparisons were subsequently performed with Bonferroni adjustment for multiple testing. Statistical significance was set at *p* < 0.05.

## Results

### Thickness variation across tooth regions

Generalized estimating equation (GEE) analysis demonstrated statistically significant differences in aligner thickness among tooth regions (Wald test, *p* < 0.001). Relative to the anterior region, all posterior regions exhibited significantly greater mean thickness values. The largest difference was observed between molars and anterior teeth (86.65 ± 1.47 µm; 95% CI: 83.77–89.53 µm), followed by canines (68.32 ± 1.79 µm; 95% CI: 64.81–71.83 µm) and premolars (44.84 ± 3.17 µm; 95% CI: 38.63–51.05 µm). These findings demonstrated a clear posterior-to-anterior gradient in thermoforming-induced thinning. Descriptive thickness values and percentage reductions across tooth regions are summarized in Table 2.

**Table 2:** Thickness distribution across tooth regions and GEE-based comparisons relative to the anterior region.

Tooth region	*n*	Mean thickness (µm)	Thickness reduction (%)	Mean difference relative to anterior ± SE (µm)	95% CI	*p*-value
Anterior	10	404.0 ± 5.9	46.8 ± 0.8	Reference	–	–
Canine	10	464.6 ± 5.3	38.9 ± 0.7	68.32 ± 1.79	64.81–71.83	<0.001
Premolar	10	443.2 ± 7.1	41.7 ± 0.9	44.84 ± 3.17	38.63–51.05	<0.001
Molar	10	481.6 ± 6.0	36.6 ± 0.8	86.65 ± 1.47	83.77–89.53	<0.001

Values are presented as mean ± standard deviation unless otherwise specified. The anterior region was used as the reference category in the GEE model. Estimated mean differences are presented as mean ± standard error (SE) with corresponding 95% confidence intervals.

### Pairwise comparisons between tooth regions

Bonferroni-adjusted pairwise comparisons derived from the GEE model demonstrated statistically significant differences between all tooth regions (*p* < 0.001). The greatest thickness difference was observed between molars and anterior teeth (86.65 ± 1.47 µm), followed by canine–anterior (68.32 ± 1.79 µm) and premolar–anterior comparisons (44.84 ± 3.17 µm). Significant differences were also identified among posterior regions, including molar–premolar, molar–canine, and canine–premolar comparisons. Detailed Bonferroni-adjusted pairwise comparisons are presented in Table 3.

**Table 3:** Pairwise comparisons of mean thickness between tooth regions based on GEE analysis.

Comparison	Mean difference ± SE (µm)	Bonferroni-adjusted *p*-value	95% CI
Canine vs. Anterior	68.32 ± 1.79	<0.001	63.60–73.04
Molar vs. Anterior	86.65 ± 1.47	<0.001	82.77–90.53
Premolar vs. Anterior	44.84 ± 3.17	<0.001	36.48–53.19
Molar vs. Canine	18.33 ± 1.31	<0.001	14.88–21.77
Canine vs. Premolar	23.48 ± 3.41	<0.001	14.49–32.47
Molar vs. Premolar	41.81 ± 0.29	<0.001	33.10–50.51

Pairwise comparisons were performed using Bonferroni-adjusted estimates derived from the generalized estimating equation (GEE) model. Values are presented as mean difference ± standard error (SE) with corresponding 95% confidence intervals.

### Site-specific thickness variation

GEE analysis demonstrated significant site-specific thickness variation within each tooth group (Wald test, *p* < 0.001). Detailed site-specific estimated mean thickness values are presented in Table 4.

**Table 4:** Site-Specific estimated mean thickness across representative teeth based on GEE analysis.

Tooth	Site	Estimated mean thickness ± SE (µm)	95% CI	*p*-value*
Anterior	FC	273.2 ± 2.93	267.46–278.94	<0.001
	FLA	425.5 ± 1.75	422.08–428.92	
	I	397.4 ± 2.18	393.12–401.68	
	PC	389.3 ± 3.46	382.51–396.09	
	PLA	493.6 ± 1.99	489.69–497.51	
Canine	FC	380.8 ± 1.85	377.18–384.42	<0.001
	FLA	472.1 ± 2.37	467.46–476.74	
	I	430.1 ± 1.55	427.06–433.14	
	PC	486.3 ± 2.29	481.81–490.79	
	PLA	551.3 ± 3.44	544.56–558.04	
Molar	FC	454.8 ± 1.36	452.13–457.47	<0.001
	FLA	441.9 ± 2.18	437.62–446.18	
	OS	550.4 ± 0.99	548.49–552.40	
	PC	455.9 ± 1.95	452.09–459.71	
	PLA	509.2 ± 1.97	505.34–513.06	
Premolar	FC	262.5 ± 2.36	257.87–267.13	<0.001
	FLA	313.3 ± 2.56	308.28–318.32	
	OS	478.7 ± 1.15	476.44–480.95	
	PC	550.5 ± 9.10	532.66–568.34	
	PLA	598.2 ± 10.39	577.83–618.57	

* *p*-values represent overall Wald tests for site-specific differences within each tooth group. Values are presented as estimated mean thickness ± standard error (SE) with corresponding 95% confidence intervals. Statistical analyses were performed using generalized estimating equation (GEE) models with Wald chi-square tests. OS values represent pooled cusp-tip measurements.

In the anterior region, the facial cervical (FC) site exhibited the lowest estimated mean thickness (273.2 ± 2.93 µm), whereas the palatal long-axis (PLA) retained the greatest thickness (493.6 ± 1.99 µm). A similar pattern was observed in canines, where FC demonstrated the lowest thickness (380.8 ± 1.85 µm) and PLA the highest (551.3 ± 3.44 µm). Overall, facial surfaces consistently exhibited lower thickness values than corresponding palatal surfaces.

In posterior teeth, site-specific differences were also significant (*p* < 0.001). In premolars, the facial cervical region showed the lowest estimated thickness (262.5 ± 2.36 µm), whereas palatal surfaces retained greater thickness values, particularly at the palatal long-axis site (598.2 ± 10.39 µm). Similarly, in molars, lower thickness values were observed at facial sites, whereas occlusal and palatal regions retained comparatively greater thickness.

#### Overall pattern of thickness distribution

Collectively, the GEE analyses demonstrated a reproducible and spatially non-uniform pattern of thermoforming-induced thickness variation characterized by progressive anterior thinning and reduced thickness at facial cervical regions.

## Discussion

The present study provides a detailed evaluation of thickness distribution in thermoformed clear aligners and demonstrates that material deformation during thermoforming results in pronounced and spatially non-uniform thinning. The findings revealed a consistent and reproducible pattern in which thickness varied systematically across both tooth regions and anatomical sites, indicating that aligner thickness is primarily governed by tooth morphology and local geometric characteristics.

A clear posterior-to-anterior gradient in thickness was observed, with molars exhibiting the greatest thickness (481.6 ± 6.0 µm) and anterior teeth showing the most pronounced thinning (404.0 ± 5.9 µm), corresponding to an overall reduction of approximately 36%–47% relative to the original material thickness. This gradient reflects differential strain during thermoforming, as posterior teeth typically present broader and flatter surfaces that undergo less stretching, whereas anterior teeth are characterized by sharper curvature and reduced surface area, leading to increased material elongation. Similar anterior–posterior trends have been reported in previous studies, where thinner aligners were consistently observed at incisors compared with molars (7, 8). These findings reinforce the role of surface curvature and crown morphology as primary determinants of thermoforming-induced deformation.

GEE-based pairwise comparisons further confirmed statistically significant differences between all tooth regions. The largest difference was identified between molars and anterior teeth (86.65 µm; 95% CI: 83.77–89.53 µm), supporting the presence of a strong spatial gradient in thermoforming-induced deformation. Collectively, these findings indicate that anatomical geometry appears to exert a major influence on thickness distribution throughout the aligner.

At the site-specific level, thickness variation was strongly dependent on anatomical location within individual teeth. The facial cervical region generally exhibited the greatest degree of thinning, particularly in anterior teeth (273.2 ± 2.93 µm) and premolars (262.5 ± 2.36 µm), whereas palatal regions retained substantially greater thickness. In contrast, palatal long-axis (PLA) sites demonstrated the greatest thickness retention across multiple tooth groups. This pattern is consistent with previous investigations demonstrating that thermoformed aligners are thinner at gingival margins than at occlusal or incisal areas (9, 10). Mechanistically, this can be explained by differences in local curvature and strain distribution. Cervical regions, particularly on the facial surface, are characterized by steep curvature and undercuts, which increase tensile stress during thermoforming and result in greater material thinning. In contrast, palatal surfaces are generally less convex and therefore undergo less deformation, allowing them to retain greater thickness.

The consistent observation that facial surfaces are thinner than their palatal counterparts further highlights the role of asymmetric geometry in thermoforming. Across both anterior and posterior teeth, facial regions demonstrated lower thickness values than corresponding palatal sites. This asymmetry may lead to heterogeneous stiffness distribution within the aligner, where thinner regions exhibit reduced resistance to deformation while thicker regions remain comparatively rigid. Such non-uniformity may potentially influence aligner adaptation and the uniformity of contact with tooth surfaces.

From a biomechanical perspective, these variations in thickness are clinically relevant. Aligner stiffness is highly dependent on thickness, with force magnitude increasing nonlinearly as thickness increases (11, 12). Consequently, localized thinning, particularly in the facial cervical region, may result in reduced force delivery and diminished control of certain tooth movements, especially those requiring precise torque expression. Conversely, relatively thicker regions, such as occlusal or palatal surfaces, may generate higher forces, potentially contributing to imbalanced force systems. Such non-uniform thickness distribution may partially explain discrepancies between predicted and achieved tooth movements reported in previous clinical studies (4, 5).

In addition to force delivery, thickness variability may also influence the mechanical behavior of aligners under functional loading. Regions with substantial thinning may be more susceptible to deformation, while thicker regions may resist deformation to a greater extent, potentially contributing to uneven stress distribution across the appliance (13). Increased thickness in occlusal regions may also contribute to occlusal interference or bite-block effects, which have been associated with transient posterior open bite during aligner therapy (14, 15). These findings suggest that non-uniform thickness distribution is not merely a manufacturing artifact but may have clinically relevant consequences.

The results of this study also have implications for aligner design and fabrication. Given that thickness distribution is strongly influenced by tooth morphology and local curvature, strategies aimed at compensating for thermoforming-induced deformation may improve biomechanical predictability. Digital modifications of virtual models or the use of advanced manufacturing techniques may help achieve a more controlled thickness profile. In particular, direct three-dimensional printing technologies offer the potential to locally modulate thickness, allowing reinforcement of regions requiring increased stiffness, such as cervical areas for improved torque control (16, 17).

In summary, thermoformed multilayer clear aligners exhibit significant and systematic non-uniform thickness distribution characterized by greater thinning in anterior regions and at facial cervical sites, and greater thickness retention in posterior and palatal regions. These patterns are primarily governed by tooth morphology and local geometry and may have important implications for aligner biomechanics, force delivery, and clinical predictability.

## Conclusion

Thermoformed clear aligners exhibited significant and non-uniform thickness variation across both tooth regions and anatomical sites. A consistent posterior-to-anterior gradient was observed, with greater thinning in anterior teeth, while site-specific analysis revealed that the facial cervical region was the most susceptible to thinning and palatal surfaces retained greater thickness. These findings indicate that thickness distribution is strongly influenced by tooth morphology and local geometric characteristics rather than being uniform across the aligner. Such non-uniform thickness distribution may influence the mechanical behavior of aligners, particularly in terms of force delivery and biomechanical predictability. Improved control of thickness distribution during fabrication may help optimize aligner performance and enhance clinical outcomes.

### Limitations

This study evaluated aligner thickness using a single thermoforming device and one multilayer material (Zendura FLX); therefore, the findings may not be directly generalizable to other materials or fabrication systems. In addition, the relatively small sample size and the *in vitro* study design may limit extrapolation of the findings to broader clinical conditions. Although multiple repeated anatomical measurements were obtained from each aligner and analyzed using generalized estimating equations (GEE), further studies with larger sample sizes and different thermoforming systems are warranted. Furthermore, thickness measurements were performed using a calibrated digital caliper rather than micro-computed tomography (micro-CT), which may limit three-dimensional characterization of thickness distribution. Future studies employing advanced imaging may provide more comprehensive evaluation of aligner thickness distribution and thermoforming-induced deformation patterns.

## Data Availability

The original contributions presented in the study are included in the article/Supplementary Material, further inquiries can be directed to the corresponding author.
